# Supplementary figures and images for: N6-methyladenosine modification and the YTHDF2 reader protein play cell type specific roles in lytic viral gene expression during Kaposi's sarcoma-associated herpesvirus infection
Source: PLoS Pathog. 2018 Apr 16;14(4):e1006995. doi: 10.1371/journal.ppat.1006995 (PMC5919695; doi:10.1371/journal.ppat.1006995)

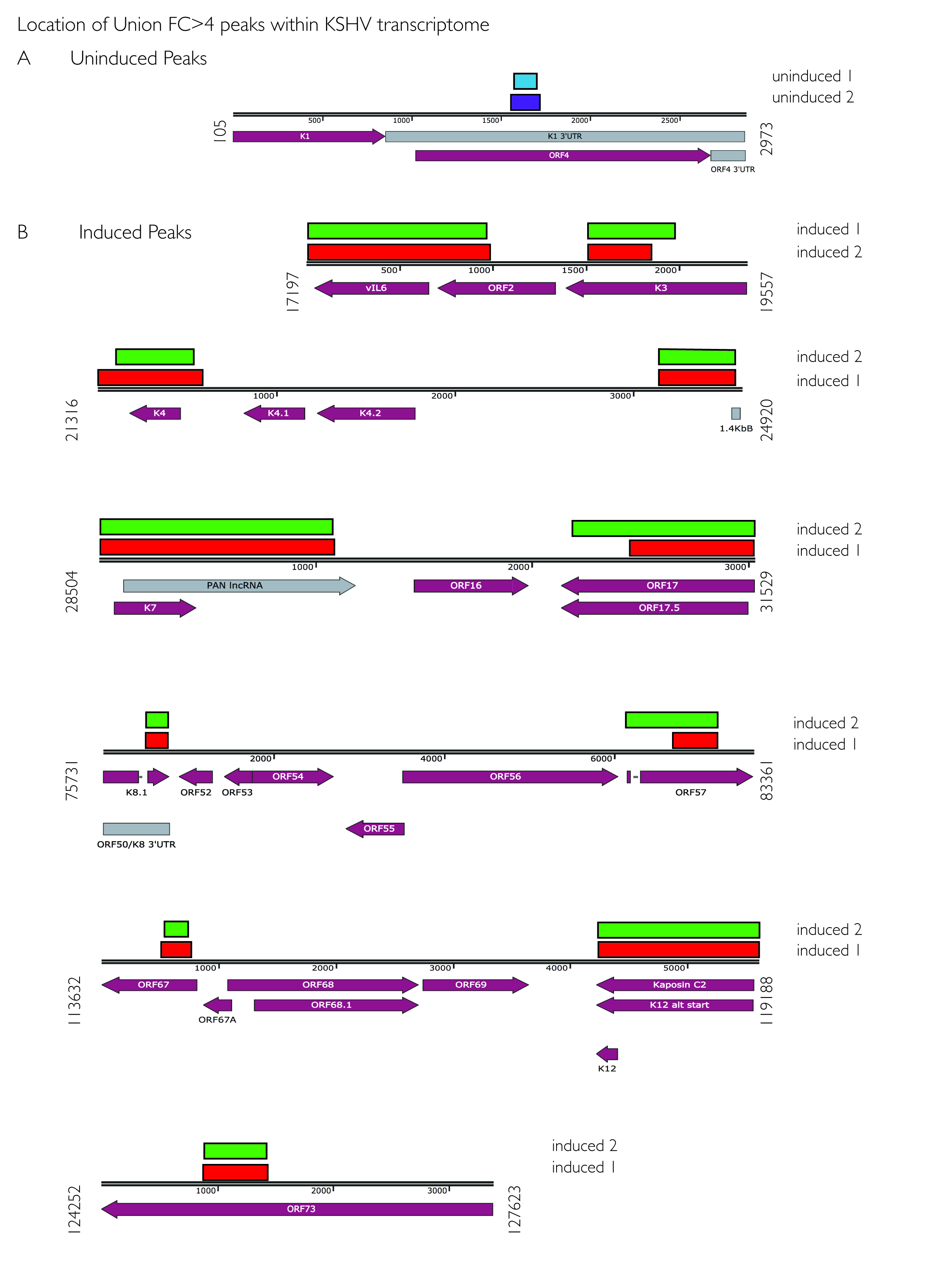

Supplement: S1 Fig — Overview of sequencing reads aligned to regions of the KSHV transcriptome containing m6A modifications. Depicted are peaks with a fold change of four or higher in both replicates, comparing reads in the m6A-IP to the corresponding input. The blue and purple bars denote the sequences encompassed by the FC>4 peaks in each uninduced replicate (A), while the red and green colored bars denote the FC>4 peaks in each induced replicate (B). In the reference transcriptome, grey bars indicate annotated 3’UTRs or ncRNAs, while burgundy arrows depict ORFs. Note that the alignment of the ORF50 induced peaks can be found in Fig 2. (TIF) [file ppat.1006995.s001.tif]

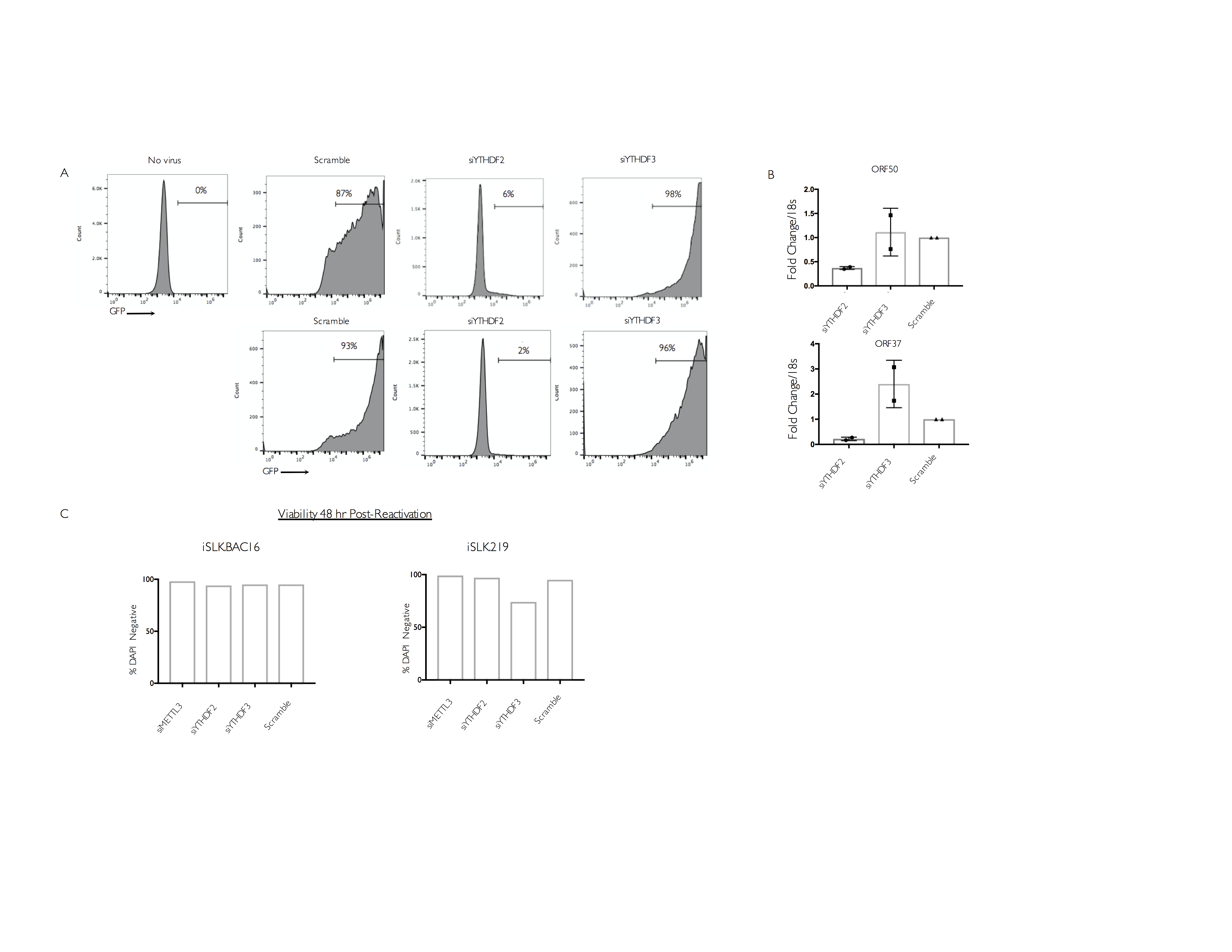

Supplement: S2 Fig — (A) Independent siRNAs against YTHDF2 and YTHDF3 yield similar results as those shown in Fig 3. Cells were transfected with control scramble (scr) siRNAs (Qiagen SI03650318) or siRNAs against YTHDF2 (Qiagen SI04174534) or YTHDF3 (Qiagen SI00764778), then reactivated for 72 hr with doxycycline and sodium butyrate. Viral supernatant was collected from the reactivated iSLK.219 cells and transferred to uninfected HEK293T recipient cells. 24 hr later, the recipient cells were analyzed by flow cytometry for the presence of GFP, indicating transfer of infectious virions. Data are from 2 independent experiments, with each replicate shown. (B) ORF50 and ORF37 gene expression was analyzed by RT-qPCR from the above cells at the time of supernatant transfer. (C) Viability of iSLK.BAC16 and iSLK.219 cells following siRNA transfection. Cells were transfected with the indicated siRNAs for 48 hr, followed by lytic reactivation with dox and sodium butyrate for 48 hr. Cells were collected and diluted 1:1 with Trypan blue prior to counting on a Countess II Automated Cell Counter. One representative experiment is shown. (TIFF) [file ppat.1006995.s002.tiff]

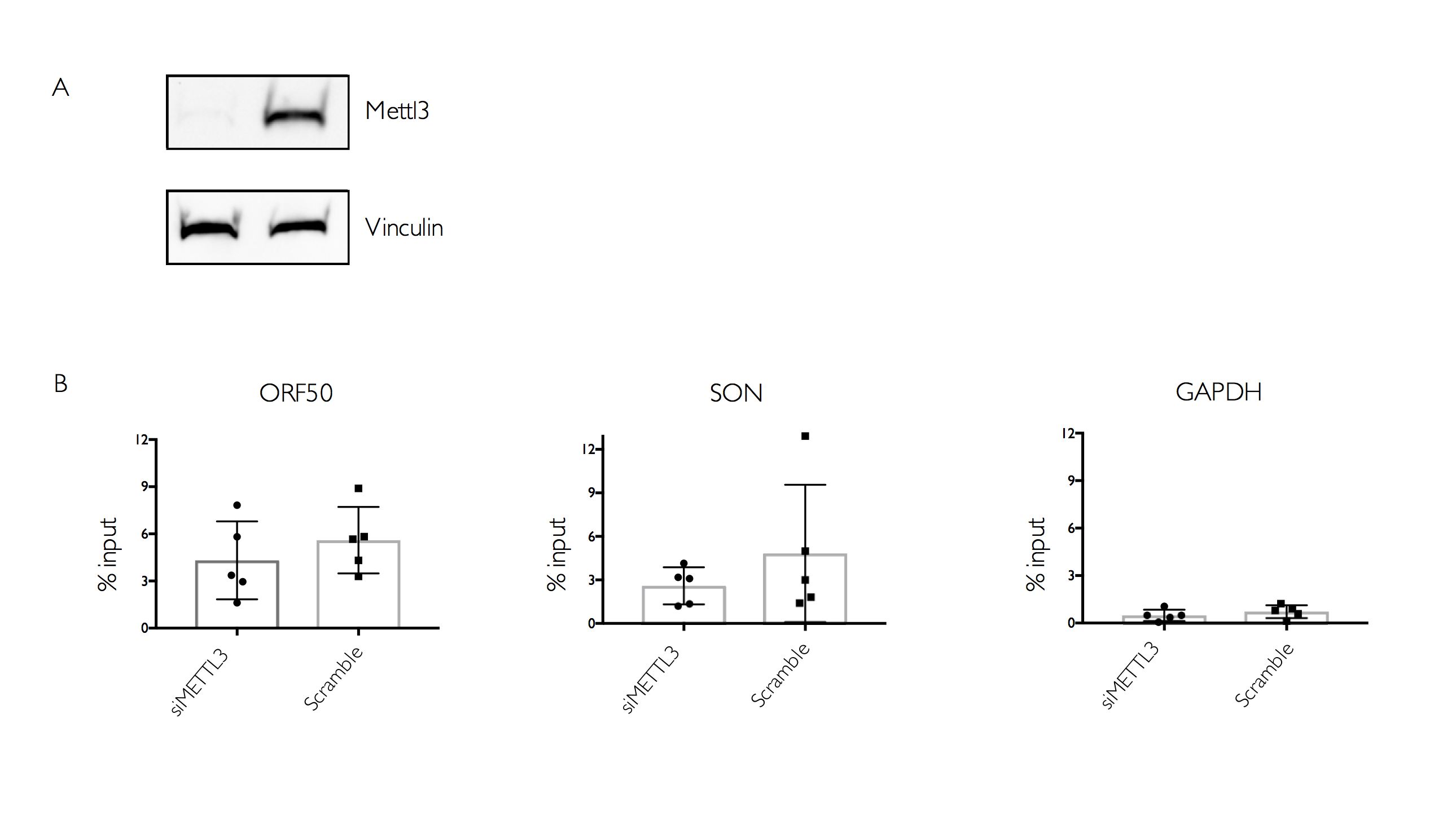

Supplement: S3 Fig — iSLK.BAC16 cells were subject to siRNA knockdown using METTL3 or control siRNA for 48 hr. Cells were reactivated for 24 hr with dox. (A) Western blot for knockdown efficiency at time of harvest. (B) Total RNA from harvested cells was then subject to m6A RIP RT-qPCR for the viral transcript ORF50 and cellular transcripts SON (m6A modified) and GAPDH (unmodified). Data shown are from 5 independent experimental replicates. (TIFF) [file ppat.1006995.s003.tiff]

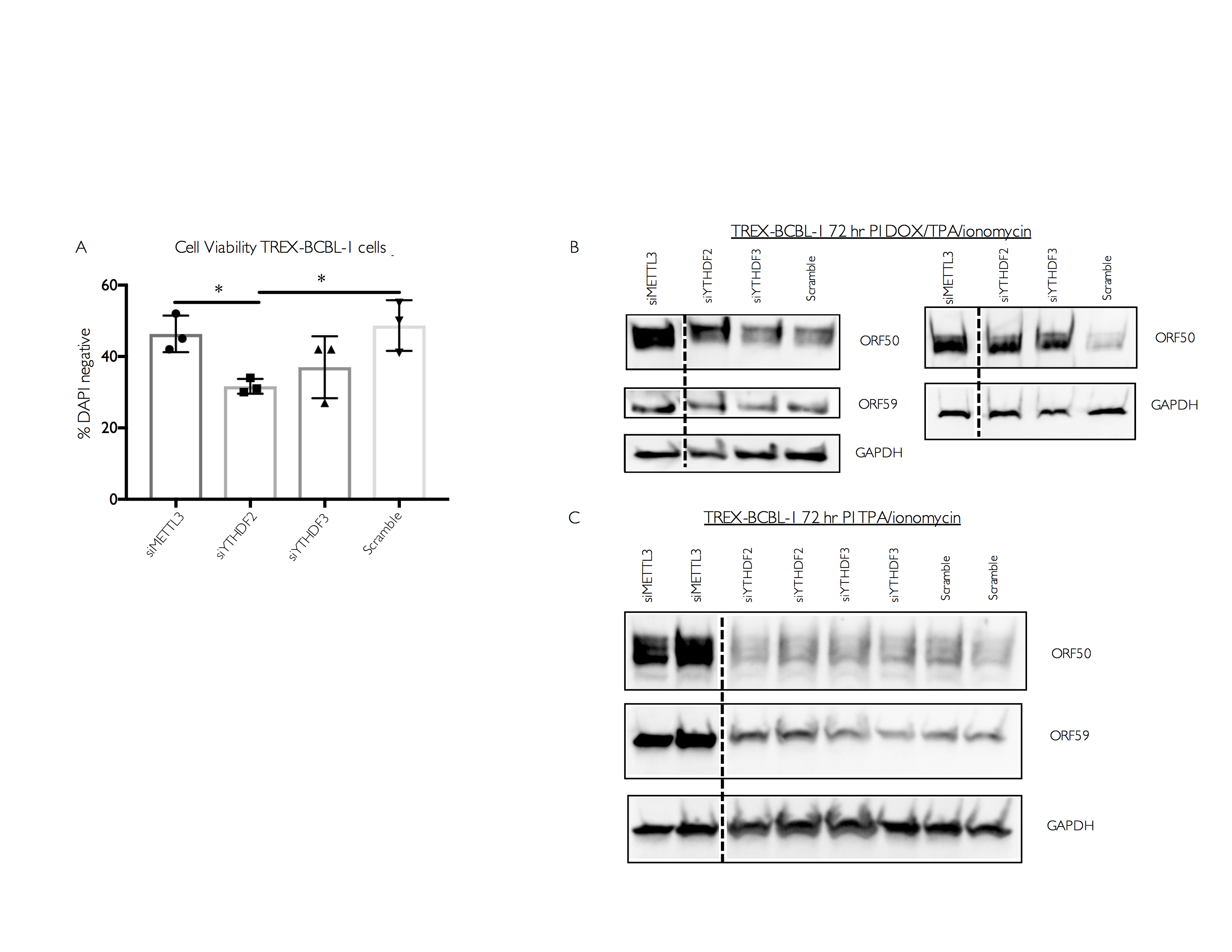

Supplement: S4 Fig — (A) Quantification of cell viability following siRNA nucleofection and reactivation in TREX-BCBL-1 cells. TREX-BCBL-1 cells were nucleofected twice with the indicated siRNAs as described in the methods, and then reactivated for 36 hr with dox, PMA and ionomycin. Cells were collected and diluted 1:1 with Trypan blue prior to counting on a Countess II Automated Cell Counter. Viability from three independent experiments is depicted in the bar graphs. Unpaired Student’s t test was used to evaluate the statistical difference between samples. Significance is shown for P values <0.05 (*). (B) Western blots from replicate experiments showing viral ORF50 and ORF59 protein levels in TREX-BCBL-1 cells treated with the indicated siRNAs and reactivated with dox, TPA, and ionomycin as described in Fig 6C. (C) Western blots showing viral ORF50 and ORF59 protein levels in TREX-BCBL-1 cells treated with the indicated siRNAs for 72 hr prior to reactivation with TPA and ionomycin. (TIFF) [file ppat.1006995.s004.tiff]

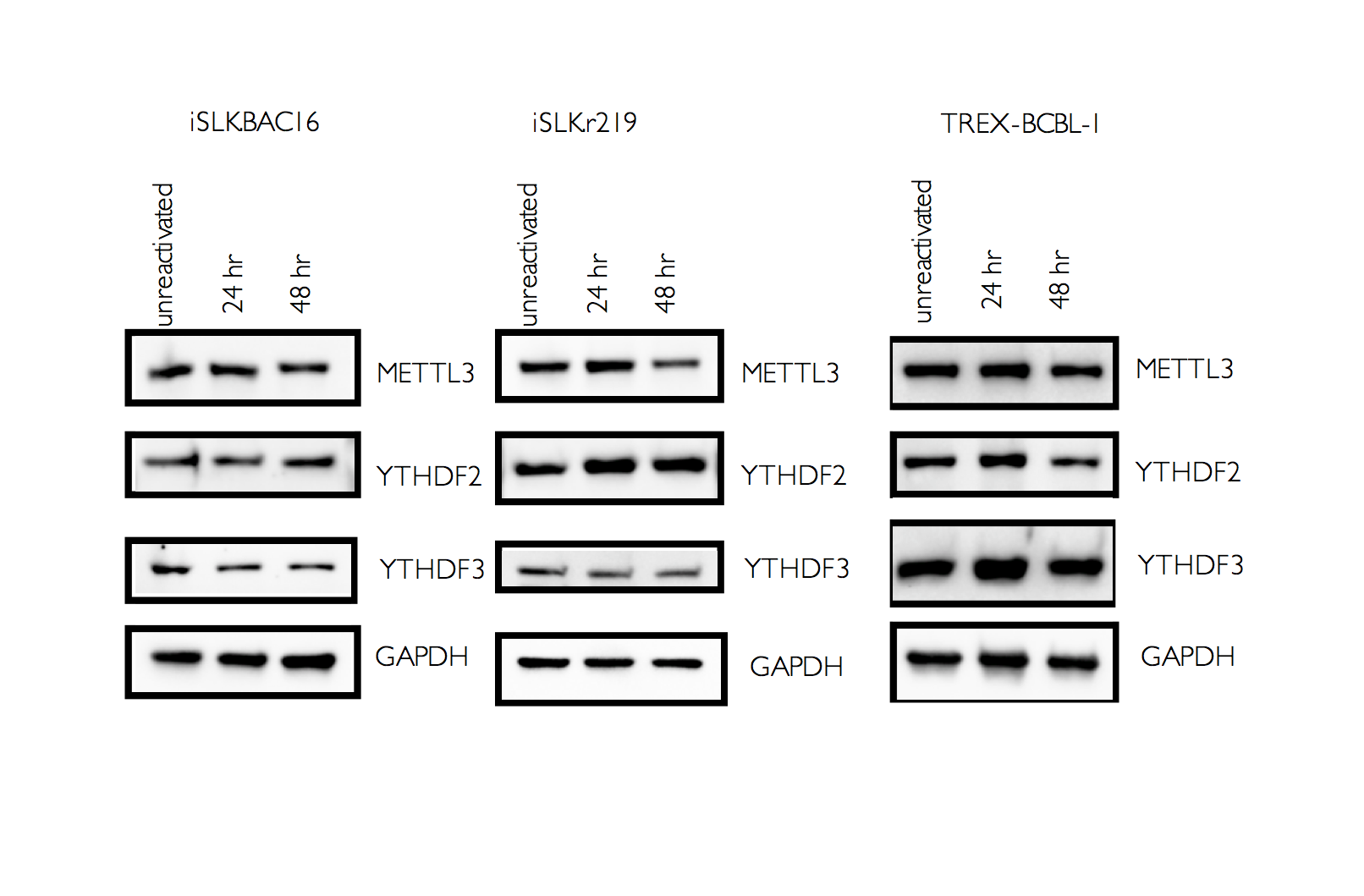

Supplement: S5 Fig — iSLK.BAC16, iSLK.219 or TREX-BCBL-1 cells were reactivated where indicated with dox for 24 or 48 hr, at which point cells were harvested and lysates were analyzed by Western blot for METTL3, YTHDF2, YTHDF3, and the GAPDH loading control. (TIFF) [file ppat.1006995.s005.tiff]
