# Supplementary material for: N6-methyladenosine modification and the YTHDF2 reader protein play cell type specific roles in lytic viral gene expression during Kaposi's sarcoma-associated herpesvirus infection
Source: PLoS Pathog. 2018 Apr 16;14(4):e1006995. doi: 10.1371/journal.ppat.1006995 (PMC5919695; doi:10.1371/journal.ppat.1006995)
Supplement: S4 Table — (DOCX) [file ppat.1006995.s009.docx]

S4. Table: List of RT-qPCR primers used in this study.

| Primer | Sequence (5’-3’) | Orientation  F: Forward  R: Reverse |
| --- | --- | --- |
| vIL6 | CGGTTCACTGCTGGTATCTG | F |
| vIL6 | CAGTATCGTTGATGGCTGGT | R |
| ORF57 | TTTGACGAATCGAGGGACGACG | F |
| ORF57 | GCAGTTGAGAACGACCTTGAGAT | R |
| ORF37 | TGGGCGAGTTTATTGGTAGTGAGG | F |
| ORF37 | CTCCACTAGACAGCAGATGTGG | R |
| K8.1 | TCCCTAAACGGGACCAGACT | F |
| K8.1 | ACCCAGAGGCAGACGTATCT | R |
| PAN | TAATGTGAAAGGAAAGCAGCGCCC | F |
| PAN | CATTTAGGGCAAAGTGGCCCGATT | R |
| vGPCR | GTGCCTTACACGTGGAACGTT | F |
| vGPCR | GGTGACCAATCCATTTCCAAGA | R |
| K1 | CCAAACGGACGAAATGAAAC | F |
| K1 | TGTGTGGTTGCATCGCTATT | R |
| GAPDH | cggagtcaacggatttggtcgtat | F |
| GAPDH | agccttctccatggtggtgaagac | R |
| ORF50 | CGCAATGCGTTACGTTGTTG | F |
| ORF50 | GCCCGGACTGTTGAATCG | R |
| ORF50 viral | GAGTCCGGCACACTGTACC | F |
| ORF50 viral | AAACTGCCTGGGAAGTTAACG | R |
| DICER | TGCTATGTCGCCTTGAATGTT | F |
| DICER | AATTTCTCGATAGGGGTGGTCTA | R |
| 18s | GTAACCCGTTGAACCCCATT | F |
| 18s | CCATCCAATCGGTAGTAGCG | R |
| LANA | TGGCCCATCTCGCGAATA | F |
| LANA | AACGCGCCTCATACGAACTC | R |
| SON | CGACAGCGCTGGAATCCTAT | F |
| SON | GCCATCAAGGGATCCACTCC | R |
